# Clinical, hormonal, and genetic characteristics of 25 Chinese patients with idiopathic hypogonadotropic hypogonadism

**DOI:** 10.1186/s12902-022-00940-9

**Published:** 2022-01-28

**Authors:** Qingxu Liu, Xiaoqin Yin, Pin Li

**Affiliations:** Department of Endocrinology, Shanghai Children’s Hospital, Shanghai Jiao Tong University, Shanghai, 200062 People’s Republic of China

**Keywords:** Idiopathic hypogonadotropic hypogonadism, Kallmann syndrome, Variant

## Abstract

**Background:**

Idiopathic hypogonadotropic hypogonadism (IHH) is a type of congenital disease caused by a variety of gene variants leading to dysfunction in the secretion of hypothalamic gonadotropin-releasing hormones (GnRHs). Clinically, IHH can be divided into Kallmann syndrome (KS) with dysosmia and normosmic idiopathic hypogonadotropic hypogonadism (nIHH) according to the presence or absence of an olfactory disorder.

**Methods:**

We retrospectively evaluated 25 IHH patients (8 KS and 17 nIHH) who were diagnosed at the Department of Endocrinology of Shanghai Children’s Hospital from 2015 to 2021. We analysed the patients’ clinical data, including their hormone levels and gene sequences.

**Results:**

All male patients exhibited small phalli, and 35% of them exhibited cryptorchidism. A significant difference was observed in the levels of dihydrotestosterone (DHT) after human chorionic gonadotropin (HCG) stimulation (*P* = 0.028) between the KS group and the nIHH group. Missense variants were the major cause of IHH, and the main pathogenic genes were FGFR1, PROKR2/PROK2, and KAl1. Nine reported and 13 novel variants of six genes were identified. De novo variants were detected in 16 IHH patients; eight patients inherited the variants from their mothers, while only three patients inherited variants from their fathers. One patient had both KAl1 and PROKR2 gene variants, and another patient had two different PROKR2 gene variants. These two patients both had the hot spot variant c.533G > C (p. Trp178Ser) of the PROKR2 gene.

**Conclusion:**

IHH should be highly suspected in patients with a small phallus and cryptorchidism. Compared with nIHH patients, KS patients exhibited a higher level of DHT after HCG stimulation. Missense variants were the major cause of IHH, and most of the inherited variants were from their mothers who exhibited no obvious clinical symptoms. We identified 9 reported variants and 13 novel variants that led to IHH. A small proportion of patients were at risk of inheriting either the oligogenic variant or the compound heterozygous variant. The hot spot variant c.533G > C (p. Trp178Ser) of PROKR2 might be involved in oligogenic inheritance and compound heterozygous inheritance. These findings provide deeper insight into the diagnosis and classification of IHH and will contribute to its clinical assessment.

## Background

Idiopathic hypogonadotropic hypogonadism (IHH), also known as congenital hypogonadotropic hypogonadism (CHH), is caused by insufficient production or secretion of hypothalamic gonadotropin-releasing hormone (GnRH) [1]. IHH can be divided into Kallmann syndrome (KS) with dysosmia and normosmic idiopathic hypogonadotropic hypogonadism (nIHH) depending on the absence or presence of olfactory disorders. The prevalence of IHH in males is approximately 1/10,000, and the ratio of males to females with IHH is 5:1 [1, 2]. Due to the low prevalence of IHH, only a few detailed studies on its basic clinical characteristics, related congenital malformations, hormone levels, and gene variants have been conducted using teenagers. Most of the reported studies mainly include Caucasians, which explains the known gene variant in 50% of those with IHH [3]; however, a large number of pathogenic genes still need to be evaluated. Previously, most patients with IHH were diagnosed and treated in late adolescence or early adulthood, as IHH was difficult to diagnose. Some studies that address the clinical and hormonal characteristics of large groups do not reveal the underlying variants. Hence, we aimed to retrospectively evaluate the clinical manifestations, genotypes, and serum hormones of 25 IHH patients in whom gene variants were identified to better understand the mechanism underlying the development of IHH.

## Patients and methods

### Patients

Informed parental consent, patient consent, and approval from the Hospital Ethics Committee were obtained prior to the initiation of the study. Twenty-five sporadic patients (aged 14 to 17.5 years) with IHH were recruited from the Department of Endocrinology, Shanghai Children’s Hospital, from 2015 to 2021. Patients who had a normal karyotype, hypothalamus and pituitary, pituitary adrenal axis, thyroid axis, and growth hormone axis were included in this study. They exhibited IHH-related variants and clinical signs of hypogonadotropic hypogonadism. IHH can be diagnosed if the luteinizing hormone (LH) is ≤8 IU/L after 60 min of gonadorelin stimulation or ≤ 4 IU/L after 60 min of triptorelin stimulation in men and when the LH is ≤6 IU/L after 60 min of the above stimulations in females [4, 5]. We investigated the clinical manifestations, related sex hormones, and gene sequences of the patients and their parents. In our study, IHH was classified as either KS or nIHH according to the absence or presence of olfactory disorders.

### External genital phenotype evaluation

We evaluated the external genital phenotypes, family history, olfactory disorders, and puberty development disorders in family members. Phallus length was compared with that of normal Chinese children. The phallus was placed in an extended state, and the length from the pubic symphysis to the top of the glans along the dorsal side of the phallus excluding the length of the foreskin was recorded as the length of the phallus. Cryptorchidism was confirmed by physical examination and ultrasound examination based on the diagnostic criteria for paediatric cryptorchidism.

### Hormonal analysis

To assess testicular function, male patients underwent human chorionic gonadotropin (HCG) stimulation. All patients underwent gonadotropin-releasing hormone (GnRH) stimulation to assess hypothalamic-pituitary-gonadal (HPG) axis function. Sex hormones, including anti-Mullerian hormone (AMH), inhibin B (INHB), oestradiol (E2), sex hormone-binding globulin (SHBG), basal testosterone (T), basal dihydrotestosterone (DHT), basal LH, peak LH, basal follicle-stimulating hormone (FSH), peak FSH, DHT after HCG stimulation, and T after HCG stimulation, were detected. Serum LH and FSH concentrations were tested using LH and FSH detection kits (Beckman Coulter) and measured with an automatic immunoluminescence analyser (Unicel DxI 800). The serum AMH and INHB levels were detected using solid-phase sandwich enzyme-linked immunosorbent assay (ELISA) kits purchased from Guangzhou Kangrun Biotechnology Co., Ltd. E2. The T and DHT levels were tested by ELISA and measured with a USA Polar ELx800 microplate reader.

### IHH gene analysis

Genomic DNA of patients and their parents was obtained from peripheral blood leukocytes via a salting out procedure. Whole exomes were amplified and sequenced to screen for variants. We searched the variant assessment database within the human gene mutation database (http://www.ncbi.nlm.nih.gov) and the ClinVar database (https://www.ncbi.nlm.nih.gov/clinvar/). PROVEAN, SIFT and Mutation Taster software were used to perform a pathogenicity analysis of amino acid changes caused by variants. The minor allele frequency (MAF) was checked for the observed variants in the Asian population. The American College of Medical Genetics and Genomics (ACMG) criteria were used for the classification of variants.

### Statistical analysis

SPSS 26.0 software (manufactured by International Business Machines Corporation) was used to analyse these data. The nonparametric data were analysed using the Mann–Whitney U test and are presented as median values. Spearman’s correlation analysis was used to perform a correlation analysis. Fisher’s test was applied to compare the incidence of clinical manifestations. A *P* value of < 0.05 was considered significant, which was indicated as follows: *P < 0.05 and **P < 0.01.

## Results

### Clinical manifestations

Of the 25 patients with IHH (23 males and 2 females), 8 had KS, and 17 had nIHH. All male patients exhibited a small phallus, and 35% of them exhibited cryptorchidism. All patients with IHH had an absence of hypospadias or ambiguous genitalia. Of the two female patients with infantile uterus and ovary, one had a cleft lip and palate, while the other was short in stature. Twenty-three male patients all had small phalli and small testes. Eight patients exhibited cryptorchidism (KS group, 2; nIHH group, 6). Six patients exhibited a short stature (KS group, 2; nIHH group, 4). Five patients developed obesity (KS group, 2; nIHH group, 3). Two patients in the nIHH group had irregular tooth alignment. No significant difference was observed in the incidence of small phallus, obesity, cryptorchidism, short stature, irregular tooth alignment, cleft palate, renal abnormalities, or syndactyly between the two groups (Table 1 and Table 2).

**Table 1:** Incidence of various clinical manifestations in patients with KS and nIHH

Clinical manifestation	KS group (8 males)	nIHH group (2 females and 15 males)	Total	***P*** value
Small phallus in males	8(100%)	15(100%)	23	>0.999
Cryptorchidism in males	2(25%)	6(40%)	8	0.657
Short stature	2(25%)	4(24%)	6	>0.999
Obesity	2(25%)	3(18%)	5	>0.999
Irregular tooth alignment	0	2(13%)	2	0.526
Cleft palate	0	1(6%)	1	>0.999
Syndactyly	0	1(6%)	1	>0.999
CHARGE syndrome	0	1(6%)	1	>0.999
Renal abnormalities	0	1(6%)	1	>0.999
Infantile uterus and ovary in females	0	2(100%)	2	>0.999

**Table 2:** Hormone levels and significant differences in male patients with KS and nIHH

Hormones	KS group (***n*** = 8)	nIHH group (***n*** = 15)	***P*** value
AMH (ng/mL)	21.3 (10–73.4)	23.05 (0.42–197.2)	0.646
INHB (pg/mL)	28.93 (4.72–73.4)	16.37 (1.71–287.8)	0.632
Oestradiol (pmol/L)	73 (73–197)	73 (73–90)	0.537
Basal LH (IU/L)	0.2 (0.1–0.45)	0.2 (0.17–0.73)	0.696
Peak LH (IU/L)	1.24 (0.31–5.86)	1.49 (0.53–4.12)	0.764
Basal FSH (IU/L)	0.54 (0.25–0.81)	0.54 (0.24–1.89)	0.740
Peak FSH (IU/L)	3.08 (1.86–5.65)	4.3 (2.28–10.81)	0.084
Basal testosterone (nmol/L)	0.35 (0.35–1.95)	0.35 (0.35–1.25)	0.363
T after HCG stimulation (nmol/L)	1.85 (0.6–7.5)	1.8 (0.6–10.1)	0.646
Basal DHT (pg/ml)	272.2 (59.92–640.1)	149.5 (20.02–359.7)	0.087
DHT after HCG stimulation (pg/ml)	306.6 (116.2–690.3)	107.4 (30.36–531.7)	0.028
SHBG (nmol/l)	42.3 (5.5–73.4)	65.9 (6.4–180)	0.104

### Hormones

**Fig. 1:**
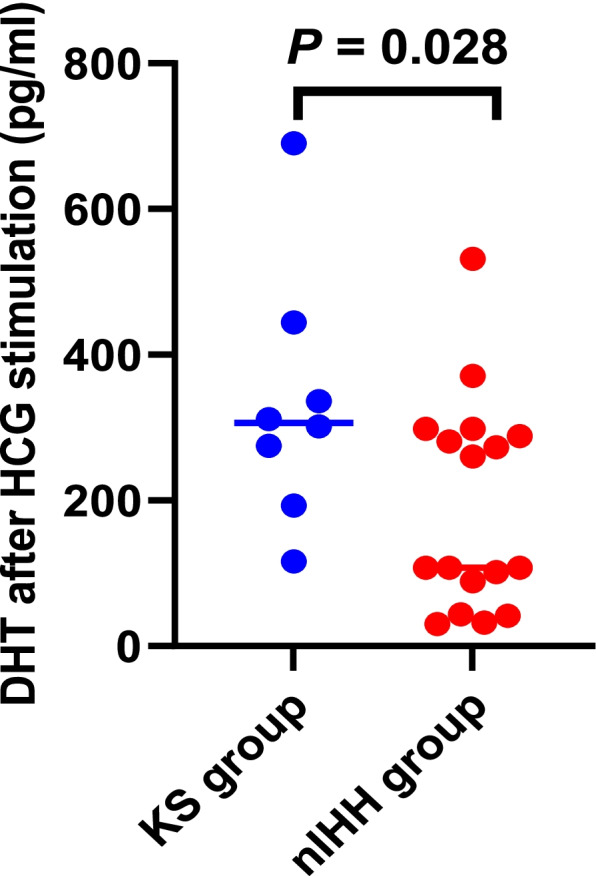
DHT levels after HCG stimulation in patients with KS and nIHH

All patients had a peak LH value of less than 6 IU/L. No significant difference between the KS and nIHH groups was observed in the levels of AMH, INHB, SHBG, basal LH, basal FSH, basal DHT, peak LH, peak FSH, or T after HCG stimulation. In contrast, a significant difference was found in the levels of DHT after HCG stimulation (*P* = 0.028) between the two groups. The data suggested that KS patients presented higher levels of DHT after HCG stimulation than did nIHH patients (Table 2 and Fig. 1).

**Fig. 2:**
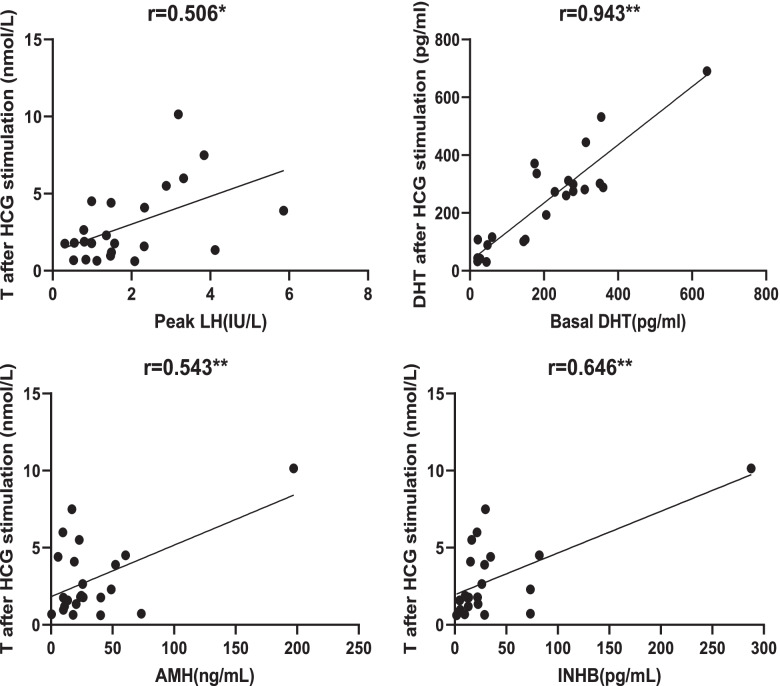
Correlation analysis of serum hormones in patients with IHH

The correlation analysis of serum sex hormone levels in IHH patients showed that DHT after HCG stimulation was positively correlated with testicular volume (*r* = 0.569, *P* < 0.01) and basal DHT levels (*r* = 0.903, *P* < 0.01). BMI was negatively correlated with peak FSH (*r* = − 0.414, *P* < 0.05) and SHBG (*r* = − 0.722, *P* < 0.01). T levels after HCG stimulation were positively correlated with AMH (*r* = 0.543, *P* < 0.01), INHB (*r* = 0.646, *P* < 0.01), and peak LH levels (*r* = 0.506, *P* < 0.05). Peak FSH was positively correlated with peak LH levels (*r* = 0.552, *P* < 0.01). A close positive correlation was observed between AMH and INHB levels (*r* = 0.943, *P* < 0.01) (Fig. 2).

### Molecular studies

The proportions of various gene variants in patients were as follows: KAl1 gene (4/25), PROKR2 (8/25), PROK2 (2/25), FGFR1 (11/25), CHD7 (1/25), and SEMA3A (1/25). Nine reported pathogenic and 13 novel variants of six genes were identified, among which the new missense variants were predicted as harmful by multiple protein function prediction software programs. De novo variants were found in 16 IHH patients; eight patients inherited the variants from their mothers, while only three patients inherited variants from their fathers, who carried heterozygous variants. Four KAl1 gene variants (two nonsense variants and two frameshift variants caused by deletion) and four FGFR1 frameshift variants (one duplication, one insertion, and two deletion) resulted in protein synthesis termination. Of the 11 (73%) FGFR1 variants, 8 were novel variants. Two novel variants in the PROK2 gene were detected in two patients, and three reported pathogenic missense variants of the PROKR2 gene were detected in eight patients. One patient had a reported pathogenic variant in the CHD7 gene, while the other patient had a novel missense variant in the SEMA3A gene. One patient had KAl1 and PROKR2 gene variants simultaneously, while the other patient had two kinds of PROKR2 gene variants. These two patients both had the hot spot variant c.533G > C (p. Trp178Ser) of the PROKR2 gene. This variant was also found in the other four patients (Table 3).

**Table 3:** Gene variants in 25 IHH patients

Case	Age (yr)	Gene	Variant	Novel	Amino acid	Pathogenicity	PROVEAN	SIFT	Mutation Taster	MAF (%)	Source of variant
M1	14.4	KAL1	c.1891C > T	N	p. Arg631Ter	P	U	U	U	na.	De novo
M2	14.3	KAL1	c.1525delA	Y	p. Ser509fs	P	U	U	U	na.	De novo
M3	14.7	KAL1	c.1267C > T	N	p. Arg423Ter	P	U	U	U	na.	Mother
M4	14.7	KAL1	c.1524delA	Y	p. Ser509fs	P	U	U	U	na.	De novo
		PROKR2	c.533G > C	N	p. Trp178Ser	VUS	−12.34	0.0	1	0.0205	De novo
M5	14	PROKR2	c.533G > C	N	p. Trp178Ser	VUS	−12.34	0.0	1	0.0205	Father
		PROKR2	c.491G > A	N	p. Arg164Gln	LP	−4.02	0.004	1	0.0028	Mother
M6	14	PROKR2	c.337 T > C	N	p. Tyr113His	LP	−4.525	0.0	1	na.	Mother
M7	15.2	PROKR2	c.533G > C	N	p. Trp178Ser	VUS	−12.34	0.0	1	0.0205	Mother
M8	14.4	PROKR2	c.533G > C	N	p. Trp178Ser	VUS	−12.34	0.0	1	0.0205	De novo
M9	15.4	PROKR2	c.533G > C	N	p. Trp178Ser	VUS	−12.34	0.0	1	0.0205	De novo
M10	14	PROKR2	c.533G > C	N	p. Trp178Ser	VUS	−12.34	0.0	1	0.0205	Father
M11	14	PROK2	c.223-4C > A	Y	U	VUS	U	U	U	na.	De novo
M12	14	PROK2	c.306G > C	Y	p. Arg102Ser	VUS	−5.46	0.0	0.999	na.	Mother
M13	15.9	FGFR1	c.761G > A	N	p. Arg254Gln	LP	−4.39	0.006	1	na.	Father
M14	15.4	FGFR1	c.963dupA	Y	p. Glu322fs	P	U	U	U	na.	De novo
M 15	14.8	FGFR1	c.1695_1696insT	Y	p. Lys566Ter	P	U	U	U	na.	De novo
M16	15	FGFR1	c.580G > T	Y	p. Gly194Cys	VUS	−6.59	0.0	1	na.	De novo
M17	14	FGFR1	c.232C > T	N	p. Arg78Cys	P	−4.82	0.0	1	na.	De novo
M18	14.9	FGFR1	c.1886 T > C	Y	p. Val629Ala	LP	−3.436	0.002	1	na.	De novo
M19	14	FGFR1	c.2008G > A	N	p. Glu670Lys	LP	−3.521	0.009	1	na.	De novo
M20	14	FGFR1	c.2147G > T	Y	p. Gly716Val	VUS	−7.549	0.007	1	na.	Mother
M21	14	FGFR1	c.1081 + 1del	Y	U	P	U	U	U	na.	De novo
M22	14	CHD7	c.5405-7G > A	N	U	P	U	U	U	na.	De novo
M23	14	SEMA3A	c.875 T > C	Y	p. Ile292Thr	VUS	−3.32	0.028	1	0.0004	Mother
F24	14.7	FGFR1	c.1974_1977del	Y	p. Asn659fs	P	U	U	U	na.	De novo
F25	17.5	FGFR1	c.75_78del	Y	p. Thr26fs	P	U	U	U	na.	Mother

*F* female, *M* male, *N* negative or no, *Y* yes, *P* pathogenic, *LP* likely pathogenic, *VUS* uncertain significance, *U* unknown, *na*. no record in gnomAD

## Discussion

IHH is a disease with genetic and clinical heterogeneity caused by dysfunction of GnRH synthesis, secretion, or action. An obvious phenotypic overlap was observed between nIHH and KS in our study. Different pathogenic genes produced similar clinical phenotypes, but the same pathogenic gene had different phenotypes. Patients with KS experience a loss or decline of olfactory function. The IHH patients in our study had a variety of reproductive and nonreproductive system phenotypes; the male patients had a small phallus, small testes, or cryptorchidism, while the female patients often exhibited a lack of secondary sexual characteristics. These clinical features often manifest after puberty; hence, it is difficult to diagnose IHH in children. Small phallus and cryptorchidism were related to the lack of androgen during the period of foetal development and were the most common reproductive system phenotypes for male teenagers, which were more serious than those of adult patients. Inguinal cryptorchidism was more common because testicular descent from the inguinal to scrotal stage is mainly driven by androgen [6]. In addition to the common olfactory abnormalities of KS, a series of related abnormalities are found in the nonreproductive systems of IHH children, including obesity, short stature, renal dysplasia, cleft palate, irregular tooth alignment, and syndactyly, which laid the foundation for the research of hormones and genes.

The sex hormone levels of the KS group and nIHH group were generally low in our study, especially the peak LH, peak FSH, and T levels after HCG stimulation. KS patients presented higher levels of DHT after HCG stimulation than did nIHH patients (median: 306.6 pg/ml vs. 107.4 pg/ml). This might be due to the higher proportion of cryptorchidism leading to the lower androgen level in the nIHH group. In addition, DHT after HCG stimulation was positively correlated with testicular volume and basal DHT. AMH and INHB could be detected in patients with IHH, which indicated normal testicular function. The testes of IHH patients responded poorly to HCG stimulation. The poor response to HCG stimulation might be related to the lack of gonadotropin stimulation for a long time. The correlation analysis of sex hormones showed that the correlation between T after HCG stimulation and AMH or INHB was higher than that with peak LH, and the correlation between AMH and INHB was extremely strong; therefore, AMH and INHB had a stronger predictive effect on T levels after HCG stimulation compared with peak LH levels.

Maintenance of the normal function of GnRH neurons requires the development, migration, secretion, and function of normal GnRH neurons, which are regulated by several types of genes [7, 8]. The KAl1 (ANOS1) gene is the first pathogenic gene of KS, which is inherited through the X-linked recessive mode. Anosmin-1 protein, encoded by the ANOS1 gene, is a type of extracellular matrix-related protein that plays an important role in the development and migration of olfactory nerve and GnRH neurons through the FGFR1 signalling pathway. Loss-of-function variants of the ANOS1 gene lead to KS, and its clinical phenotypes are relatively severe, including complete loss of puberty, infertility, cryptorchidism, and small phallus. In our study, c.1891C > T(p. Arg631*) and c.1267C > T(p. Arg423*) were the two reported variants [9, 10], while c.1525delA(p. Ser509fs) and c.1524del A(p. Ser509fs) were the two novel variants, which led to KS with small phallus, cryptorchidism, and obesity. Four kinds of KAl1 gene variants resulted in the termination of protein synthesis, the production of truncated protein, or the activation of nonsense-mediated mRNA degradation, which destroyed the integrity of the protein structure and led to the loss of protein function.

Prokineticin-2 (PROK2) is a protein that plays an important role in the development of olfactory nerve and GnRH neurons and the regulation of physiological rhythm through its receptor PROKR2. Meanwhile, KS patients present with homozygous, compound heterozygous, and heterozygous gene variants in the PROKR2 and PROKR2 genes, which can be passed down through autosomal dominant or oligogenic inheritance [11]. In our study, 40% of patients developed PROK2/PROKR2 variants, which was significantly higher than the 9% in the Caucasian population [12]. Most of them were missense variants in patients with a nonobvious phenotype [13]. In our study, only one patient had a splicing mode variant; the rest of the patients had missense variants. Some patients exhibited olfactory dysosmia and obesity, which was consistent with the clinical manifestations of extreme obesity in patients with PROKR2 variants reported in previous studies [14]. Two novel variants of the PROK2 gene were found in patients with anosmia, obvious small phallus, and low levels of sex hormones. c.223 − 4C > A might affect the normal splicing of exons in the PROK2 gene, and the novel variant c.306G > C (p. Arg102Ser) was predicted to be harmful by multiple software programs. A few missense variants were detected in patients with a PROK2 gene, and most of the missense variants recorded in the ClinVar database were pathogenic. Three kinds of missense variants in the PROKR2 gene were found in eight patients. c.337 T > C (p. Tyr113His) significantly decreased the receptor expression level and reduced intracellular calcium mobilization, resulting in protein instability and poor biological function [13]. c.491G > A (p. Arg164Gln) destroyed the interaction between the IL2 domain and G-protein, inhibited Gq-protein signal activity, and weakened G protein-coupled receptors [11]. The hot spot variant c.533G > C (p. Trp178Ser) was found in six patients and located in the transmembrane domain of the protein, which could significantly reduce the release of ionized calcium and the signal activity [15].

The FGFR1 gene is expressed in many tissues and plays an important role in the development of embryonic olfactory nerve and GnRH neurons mainly through the FGF/FGFR1 signalling pathway [16]. FGFR1 signalling is essential for the migration, secretion, or survival of hypothalamic GnRH neurons and is widely expressed in the nervous and skeletal systems. The FGFR1 gene is inherited through an autosomal dominant mode [17]. Loss of function can lead to both nIHH and KS, and more than 200 variants of the FGFR1 gene have been found in patients with IHH [18]. The FGFR1 gene had the highest variant frequency, approximately 44% in our study, which was higher than that (10%) in the Caucasian population [19]. Its variant can cause cleft lip and palate, short stature, and bone dysplasia [20, 21]. Among the 11 FGFR1 variants reported in our study, c.761G > A (p. Arg254Gln), c.232C > T (p. Arg78Cys), and c.2008G > A (p. Glu670Lys) were found to be pathogenic variants [5, 22]. Among the other eight novel variants, c.963dupA (p. Glu322fs), c.1695_ 1696insT (p. Lys566*), c.1974_ 1977del (p. Asn659fs), and c.75_ 78del (p. Thr26fs) caused the termination of protein synthesis, the production of truncated protein, or activation of nonsense-mediated mRNA degradation, which destroyed the structural integrity of the FGFR1 protein and led to the loss of FGFR1 protein function. The software predicted that the variant c.1081 + 1del would destroy the highly conserved donor splicing site activity in exon 8 and then lead to abnormal splicing, resulting in changes in the function of the protein encoded by the gene. Amino acid conservation analysis showed that the wild-type amino acid Val629 was conserved in 100 vertebrates tested, which indicated that the variant c.1886 T > C (p. Val629Ala) might not be tolerated and has adverse effects on the structure and function of the protein. In addition, the c.580G > T (p. Gly194Cys) and c.2147G > T (p. Gly716Val) variants were identified by the software as harmful. In previous studies, the phenotype of the FGFR1 gene was not completely dominant, and most of the variants were inherited from a normal father or mother. However, most of the patients in our study had de novo variants; one patient inherited the variants from his father, while the other patient inherited the variants from her mother. Two female patients had a frameshift variant of the FGFR1 gene, which showed an infantile uterus and ovary. In the group with a nonreproductive phenotype, variants in the FGFR1 gene were found in one patient with cleft lip and palate, which was consistent with the report of a previous study [20]. Another patient presented with a renal cyst and short stature. Therefore, anosmia, sexual dysplasia, irregular tooth alignment, cleft lip and palate, syndactyly, and renal abnormalities were common phenotypes of IHH patients with FGFR1 gene variants.

The CHD7 gene is located on chromosome 8q12.1 and is autosomal dominant, encoding chromosomal helicase DNA-binding protein 7. It has low to moderate expression in all tissues, including the lesion sites of KS: olfactory bulb, olfactory tract, and hypothalamus. CHARGE syndrome includes a series of organ and system abnormalities [23]. Both KS and CHARGE syndrome can be characterized by anosmia and hypogonadotropic hypogonadism, which means that KS is part of the phenotypic spectrum of CHARGE syndrome. The greater the expression of the CHD7 gene is, the more obvious the clinical features of patients with IHH, especially the symptoms of cryptorchidism, small phallus, and hypospadias in childhood [24]. Only one patient had a hot spot of intron variant c.5405 − 7G > A. In our study, it was reported to be pathogenic [25]; this finding was consistent with the results of two previous studies, which indicated that the CHD7 variant was not the primary cause of KS because only 3–5% of nIHH/KS patients had a CHD7 variant [23, 26].

SEMA3A is a reported pathogenic gene of IHH that is inherited through an autosomal dominant mode [27]. SEMA3A can affect the function of the hypothalamus pituitary gonad axis by regulating the differentiation and maturation of nerve cells, either alone or in combination with other factors (such as FGFR1) [28]. These transcription factors are widely expressed in the nervous system, and their functional defects can cause a nonreproductive phenotype, such as short stature, mental retardation, obesity, and abnormal vision. One patient presented small phallus, cryptorchidism, and irregular tooth alignment and had a novel missense variant c.875 T > C (p. Ile292Thr) in the SEMA3A gene, which was identified by software as harmful.

We described the clinical characteristics of Chinese IHH patients and the variant frequency of known pathogenic genes. We found pathogenic variants in six genes and expanded the expression profile of IHH pathogenic genes. An increasing number of scholars agree that GnRH deficiency is not strictly a single-gene disease but may be a double-gene or oligogenic genetic disease. Approximately 11.3% of patients with IHH carry two or more pathogenic gene variants, and the genetic model of oligoenes provides new insights [29]. In addition, most patients with IHH have heterozygous gene variants, and these variants are inherited from parents with a normal phenotype [30]. One patient had a novel de novo variant of KAl1 (c.1524delA, p. Ser509fs) and a hot spot variant of PROKR2 (c.533G > C, p. Trp178Ser) simultaneously. However, the clinical phenotype caused by the two gene variants was not more serious than that caused by a single gene variant because the patient manifested small phallus and small testes without a nonreproductive phenotype. In addition, one patient had two previously reported variants in the PROKR2 gene, namely, c.491G > A (p. Arg164Gln) and c.533G > C (p. Trp178Ser). The patient’s phenotype was relatively severe, and he had a short stature without obvious small phallus and small testes. These results indicated that the hot spot variant c.533G > C (p. Trp178Ser) of the PROKR2 gene might be involved in oligogenic inheritance and the complex heterozygous pathogenesis of IHH genes.

In this study, we identified nine reported gene variants, and we detected 13 novel variants: c.1525del A(p. Ser509fs) and c.1524del A(p. Ser509fs) variants in the KAl1 gene; c.223 − 4C > A and c.306G > C(p. Arg102Ser) variant in the PROK2 gene: c.963dup A (p. Glu322fs), c.1695_1696insT(p. Lys566Ter), c.580G > T(p. Gly194Cys), c.1886 T > C(p. Val629Ala), c.2147G > T(p. Gly716Val), c.1081 + 1del, c.1974_ 1977del (p. Asn659fs), and c.75_ 78del (p. Thr26fs) variants in the FGFR1 gene; and c.875 T > C (p. Ile292Thr) variant in the SEMA3A gene. The 13 novel variants identified in our study cause different types of IHH. These findings will provide deeper insight into the diagnosis of IHH and contribute to its clinical assessment.

The findings of this study have to be seen in light of some limitations. It is difficult to obtain samples for rare diseases, and the significant difference in hormone levels might be affected by the small sample size in our study. All patients in our study were sporadic, and there were no familial patients. Due to the limitations of genetic diagnosis, although many genes have been found to be associated with GnRH deficiency, the genetic basis of more than half of patients with IHH remains unknown.

## Conclusions

Abnormalities in the KAl1, PROKR2, PROK2, FGFR1, CHD7, or SEMA3A genes could cause IHH with or without nonreproductive manifestations. IHH should be highly suspected in patients with small phallus and cryptorchidism; moreover, nIHH was more common than KS. We demonstrated that nIHH and KS patients could not be distinguished based on the levels of their basal hormones, peak hormones after GnRH stimulation, or T after HCG stimulation. However, KS patients exhibited higher levels of DHT after HCG stimulation. We identified 9 reported variants and 13 novel variants of six genes that could result in IHH with different symptoms. A small proportion of patients might be affected by oligogenic inheritance or compound heterozygous inheritance. These findings provide deeper insight into the diagnosis of IHH and will contribute to its clinical assessment.

## Data Availability

All data generated or analysed during this study are included in the published article. The datasets used and/or analysed during the current study are available from the corresponding author upon reasonable request.

## Abbreviations

KS: Kallmann syndrome
IHH: Isolated hypogonadotropic hypogonadism
nIHH: Normosmic idiopathic hypogonadotropic hypogonadism
CHH: Congenital hypogonadotropic hypogonadism
INHB: Inhibin B
SHBG: Sex hormone-binding globulin
LH: Luteinizing hormone
FSH: Follicle-stimulating hormone
T: Testosterone
DHT: Dihydrotestosterone
E2: Oestradiol
HCG: Human chorionic gonadotropin
AMH: Anti-Mullerian hormone
GnRH: Gonadotropin-releasing hormone
HPG: Hypothalamic-pituitary-gonadal
MAF: Minor allele frequency
ACMG: American College of Medical Genetics and Genomics
