# Correction to: Clinical, hormonal, and genetic characteristics of 25 Chinese patients with idiopathic hypogonadotropic hypogonadism

**DOI:** 10.1186/s12902-022-00999-4

**Published:** 2022-04-02

**Authors:** Qingxu Liu, Xiaoqin Yin, Pin Li

**Affiliations:** Department of Endocrinology, Shanghai Children’s Hospital, Shanghai Jiao Tong University, Shanghai, 200062 People’s Republic of China

**Correction to: BMC Endocr Disord 23, 30 (2022)**

10.1186/s12902-022-00940-9

Following publication of the original article [1], two minor errors were found in the 12th row of the right column on page 5 and the first row of Table 3 on page 6, ‘c.1891C>T’ was mistakenly written as ‘c.1897C>T’.

The original paper has been updated.
